# Supplementary material for: Content-rich biological network constructed by mining PubMed abstracts
Source: BMC Bioinformatics. 2004 Oct 8;5:147. doi: 10.1186/1471-2105-5-147 (PMC528731; doi:10.1186/1471-2105-5-147)
Supplement: Additional File 2 — The original results of the above study (non-essential files are deleted to keep the file size under the limit set by BMC bioinformatics). [file 1471-2105-5-147-S2.bz2 › chilibotAdditionalFile2/dip05/487ID9463416E10586/html/BLR1_CXCL13.html]

 


 **BLR1** and **CXCL13** 
  
Found 38 abstracts in PubMed, retrieved 05.  
 

 What does Google say? 
 PDF only 
| .edu only 

---

**Interactive relationship** (e.g. stimulation, inhibition, etc)

**Neutral relationship**- CD3 CD4 IL 7Ralphahi cells express the chemokine receptor CXCR5  [ **BLR1** ]  and might be attracted by its ligand  **CXCL13** , which is secreted by mesenchymal cells.  Ref: 12732661 J Exp Med, 2003
- In low grade CBCL, as compared with high grade CBCL, the intensity of CXCR5  [ **BLR1** ]  expression on neoplastic CD22 cells was lower than that of BCA 1  [ **CXCL13** ] .  Ref: 12855271 Eur J Cancer, 2003
- BCA 1  [ **CXCL13** ]  and CXCR5  [ **BLR1** ]  were constantly coexpressed by CD22 B cells, while CD35 follicular dendritic cells coexpressed BCA 1  [ **CXCL13** ]  in PCBCL cells only.  Ref: 12855271 Eur J Cancer, 2003

**Non-interactive relationship** (e.g. studied together, co-existance, homology, etc.)

- Reverse transcriptase polymerase chain reaction was used to detect chemokine transcripts of CXCR4, CXCR5  [ **BLR1** ] , BLC  [ **CXCL13** ] , and SDF 1 in PIOL and retinal pigment epithelium RPE cells after microdissection either by laser capture Arcturus or by manual dissection from frozen sections.  Ref: 12578791 Ophthalmology, 2003
- We analysed the immunophenotypic and molecular expression of BCA 1  [ **CXCL13** ]  B cell specific chemokine and CXCR5  [ **BLR1** ]  BCA 1  [ **CXCL13** ]  receptor in normal skin and different cutaneous lymphoproliferative disorders cutaneous T cell lymphoma CTCL.  Ref: 12855271 Eur J Cancer, 2003
- The sections were evaluated using immunohistochemistry avidin biotin complex immunoperoxidase technique for CXCR4, CXCR5  [ **BLR1** ] , BLC  [ **CXCL13** ] , and SDF 1 to detect the expression and location.  Ref: 12578791 Ophthalmology, 2003
- Follicular B helper T T FH cells make up a CD4 memory T cell subset with unique migration properties, defined by the expression of the chemokine receptor CXCR5  [ **BLR1** ]  and responsiveness to the CXCR5  [ **BLR1** ]  selective chemokine BCA 1  [ **CXCL13** ] , which is produced in B cell follicles.  Ref: 12527215 Immunol Lett, 2003
- Expression of chemokine receptors, CXCR4 and CXCR5  [ **BLR1** ] , and chemokines, BLC  [ **CXCL13** ]  and SDF 1, in the eyes of patients with primary intraocular lymphoma.  Ref: 12578791 Ophthalmology, 2003
- BCA 1  [ **CXCL13** ]  and CXCR5  [ **BLR1** ]  were constantly expressed in CBCL and PCBCL, but not in normal skin and CTCL.  Ref: 12855271 Eur J Cancer, 2003
- **CXCL13** , SCYB13  [ **CXCL13** ]  and stromal cell derived factor 1 SDF 1, CXCL12, SCYB12 are chemotactic for human B cells, and their ligands CXCR4 and CXCR5  [ **BLR1** ]  are differentially expressed on B cells, including malignant B cells.  Ref: 12578791 Ophthalmology, 2003
